# Interventions for Perinatal Depression and Anxiety in Fathers: A Mini-Review

**DOI:** 10.3389/fpsyg.2021.744921

**Published:** 2022-01-20

**Authors:** Andre L. Rodrigues, Jennifer Ericksen, Brittany Watson, Alan W. Gemmill, Jeannette Milgrom

**Affiliations:** ^1^Parent-Infant Research Institute, Heidelberg Repatriation Hospital, Heidelberg Heights, VIC, Australia; ^2^University of Melbourne School of Psychological Sciences, Parkville, VIC, Australia

**Keywords:** postnatal, depression, anxiety, mental health, digital interventions, treatment, psychological distress, father

## Abstract

**Background and Objectives:**

Up to 10% of fathers experience perinatal depression, often accompanied by anxiety, with a detrimental impact on the emotional and behavioural development of infants. Yet, few evidence-based interventions specifically for paternal perinatal depression or anxiety exist, and few depressed or anxious fathers engage with support. This mini-review aims to build on the evidence base set by other recent systematic reviews by synthesising more recently available studies on interventions for paternal perinatal depression and anxiety. Secondarily, we also aimed to identify useful information on key implementation strategies, if any, that increase the engagement of men.

**Methods:**

We drew upon three major previous systematic reviews and performed an updated search of PubMed/Medline; Psycinfo; Cochrane Database; Embase and Cinahl. The search was limited to trials, feasibility studies or pilot studies of interventions published between 2015 and 2020 that reported on fathers' perinatal mental health. We included psychological, educational, psychosocial, paternal, couple-focused, or group therapies, delivered face-to-face, via telephone and/or online that reported on either paternal depression, anxiety or both.

**Results:**

Eleven studies satisfied search criteria (5 of which were not included in previous reviews). The majority were randomised controlled trials. Most interventions incorporated counselling, therapy or psychoeducation and took an indirect approach to perinatal mental health through antenatal or postnatal education and were couple-focused. No studies reported a presence of diagnosed depression or anxiety at baseline, although five studies reported a positive effect on sub-threshold symptoms.

**Discussion:**

There was some evidence that these approaches may be useful in the initial engagement of fathers with perinatal supports and improve depression and anxiety scores. No studies targeted the explicit treatment of clinically depressed or anxious men, and this remains the most substantial gap in the peer-reviewed evidence base. Our results highlight the need to deliver perinatal interventions specifically designed for men and evaluate them in populations with clinical levels of depressive and anxious symptomatology.

## Introduction

The transition to fatherhood can present as a fundamental shift in a man's life. Along with the traditional challenges of learning new skills and knowledge, changes in personal identity, the couple relationship and financial commitments may lead to new fathers being overwhelmed by feelings of confusion, exhaustion, helplessness, loneliness and feeling trapped (Rowe et al., 2013). Consequently, these factors can culminate in increased vulnerability to depression and anxiety.

Approximately 10% of new fathers experience significant depression in the perinatal period with debilitating symptoms of depression commonly including lowered mood, loss of interest or enjoyment, difficulty sleeping, changes in appetite and weight, feelings of worthlessness, and thoughts of self-harm (Goodman, 2004). Depression is often accompanied by significant anxiety (Tohotoa et al., 2012).

Even more are likely to suffer from milder symptoms of depression and anxiety and also a range of negative emotions such as anger, worry, confusion, and irritability (Cameron et al., 2016). Some may resent the constant needs of a new baby and their partner's preoccupation with the baby, including breastfeeding (Earls et al., 2019; Eddy et al., 2019). Depressed fathers are more likely to engage in substance abuse and family violence than non-depressed fathers (Earls et al., 2019). There is a complex relationship between depression in couples: living with a depressed partner may also exacerbate men's mental health problems and vice versa (Goodman, 2004). Depression and anxiety not only have a serious impact on men's lives in the perinatal period, but also on the emotional and behavioural development of their infants and family's functioning (Ramchandani et al., 2008).

Whilst prevalence estimates vary widely (Tuszyńska-Bogucka and Nawra, 2014; Nath et al., 2016; Paulson et al., 2016; Carlberg et al., 2018), depending on the sample surveyed and the measurement instruments used, there is growing evidence that paternal postnatal depression presents as a significant economic burden on the Australian healthcare system (PANDA, 2012). Interventions targeting paternal perinatal depression are consequently essential not only for the well-being of new fathers and their families, but to address the wider socio-economic burden.

Typically, interventions, programs and clinical health services have targeted the amelioration of maternal perinatal mental health difficulties such as depression and anxiety (Milgrom and Gemmill, 2015). We have previously reported on the efficacy of cognitive behaviour therapy (CBT) for both antenatal (Milgrom et al., 2015a) and postnatal (Milgrom et al., 2015b) depression in women using a range of delivery modes, including digital interventions (Milgrom et al., 2016). The efficacy of prevention and treatment programs for women, delivered antenatally and postnatally, has been the subject of 3 Cochrane systematic reviews (Dennis and Hodnett, 2007; Dennis et al., 2007; Dennis and Dowswell, 2013).

Significantly less is known about the likely effectiveness of interventions or programs targeting paternal perinatal mental health difficulties. In recent years, three major systematic reviews have reviewed the evidence for perinatal interventions for fathers. Twenty-six articles in total were identified by these reviews: Rominov et al. (2016), Suto et al. (2017), and Goldstein et al. (2020). Rominov et al. aimed to include a wide range of study designs and any interventions targeting fathers' mental health in the perinatal period and thus applied the broadest inclusion criteria of these major reviews. However, Suto et al. focussed more specifically on the effects of prenatal childbirth education on paternal postnatal mental health, while Goldstein et al. limited their review to randomised controlled trials (RCTs) exclusively for paternal perinatal depression.

Rominov et al. identified 11 articles and found that psychosocial interventions and massage-technique interventions, but not couple-based interventions, showed some significant effects on depression and anxiety symptoms benefitting men. Rominov et al. concluded that there was a need for improved methodological quality in this field and that none targeted active treatment of clinical levels of psychological distress in perinatal fathers.

Suto et al. identified 11 studies addressing the impact of prenatal education on paternal postnatal mental health and the couple relationship. It was concluded that there was insufficient evidence to suggest prenatal childbirth education for partners of pregnant women protects against paternal postnatal mental health difficulties (including depression and anxiety). However, the mental health of perinatal fathers was highlighted as important to maternal and perinatal healthcare.

Goldstein et al. found that of 14 RCTs identified, only three found an effect on paternal depression scores. None of the included interventions exclusively focussed on paternal perinatal depression but targeted the couple or infant relationship, with participating fathers not required to meet criteria for depression at baseline (and fathers did not undergo a clinical interview for diagnosis). Goldstein et al. highlighted the lack of active treatments for diagnosed cases of paternal perinatal depression as a major gap in perinatal mental health service provision and the methodological heterogeneities in reviewed studies regarding depression measures and follow-up.

A further issue is that very few depressed new fathers seek help or engage with currently available treatments (Olds et al., 2007; Fletcher et al., 2015). Mental health problems in new fathers can therefore go largely unacknowledged and untreated (BeyondBlue, 2018).

The aim of this mini-review is therefore to provide an updated narrative synthesis of recent, peer reviewed articles that report on experimental or quasi-experimental studies of interventions measuring perinatal depression or anxiety in fathers, whether as an intervention target, or as a primary or secondary outcome. We aim to review articles between 2015 and 2020 and identify both those included in previous reviews and new evidence. This review will provide a 5-year update of studies in this broad area which includes those that may have been published close to or after the most broadly inclusive systematic review, by Rominov et al., but which may have fallen outside of the later, more focused searches, applied by Suto et al. and by Goldstein et al. Outside the scope of previous reviews, we also aimed to identify any useful information on key implementation strategies, if any, employed to increase the engagement of men in such interventions.

## Methods

This mini-review was informed by the Preferred Reporting Items for Systematic Reviews and Meta-Analyses (PRISMA) guidelines (Moher et al., 2009). While not all PRISMA items were applied, the key principles were used to provide a structure for reporting our methods and results.

### Eligibility Criteria

This review was limited to peer-reviewed studies written in English, published in peer-reviewed journals. Articles published between 2015 and 2020 that report trials, feasibility studies or pilot studies of interventions that reported on fathers' depression or anxiety, from conception to 1 year postpartum, were included. Interventions or programs were defined broadly to include psychological, educational, psychosocial, or group therapies, delivered face-to-face, via telephone and/or online with men individually or the couple dyad.

### Information Sources

Seven databases were searched to identify recent peer-reviewed articles reporting on interventions designed to treat or prevent perinatal depressive or anxious symptoms in fathers. Ovid MEDLINE®, OVID Embase Classic + Embase, OVID Cochrane Central Register of Controlled Trials, OVID Cochrane Database of Systematic Reviews, OVID PsycINFO, EBSCO CINAHL, and PubMed. Relevant keywords and subject headings for pregnancy, birth, infancy, fathers, male parents, as well as depression and anxiety were utilised for each database.

### Search Strategies

There were two search strategies.

1. The studies identified by Rominov et al., Suto et al. and by Goldstein et al. were re-assessed for inclusion against the criteria for the current mini-review (see below), including a 5-year publication date range;
2. A new database search was run in February of 2021 including only articles published 2015–2020. We aimed to capture a comprehensive 5-year update including studies that may have been published close to or subsequent to the review published by Rominov et al., but which may have fallen outside of the inclusion criteria of Suto et al. and Goldstein et al.

Key search terms in all database searches included: (paternal^*^ or father^*^ or fatherhood^*^ or paternity^*^), (postnatal or antenatal or perinatal), (depression or anxiety or mental health or psychological distress), (treatment^*^ or prevention^*^), (programs^*^ or intervention^*^ or management) in titles and abstracts of peer-reviewed articles. The authors (JM, AG, JE, and AR) agreed upon final search terms.

### Selection Process

After duplicate citations were removed and titles and abstracts were screened, full-text articles were reviewed for eligibility by two authors (AR and JE). Two authors (AR and JE) examined the full texts of potential articles to determine eligibility for inclusion in the systematic review. Discrepancies were resolved by consensus by all the authors.

### Data Collection and Synthesis Methods

Data from the studies was manually collated into matrices in Microsoft Excel to enable a comparison of the studies aims, samples, measures of depression and anxiety, methodologies, results, and conclusions. Data extraction was completed by two reviewers (AR and JE) independently. Data from the studies were not able to be synthesised using a meta-analysis due to the heterogeneity of the methodologies adopted by the studies. Instead, a narrative synthesis was used to appraise and summarise the key findings for the included studies.

## Results

### Study Selection

**Figure 1:**
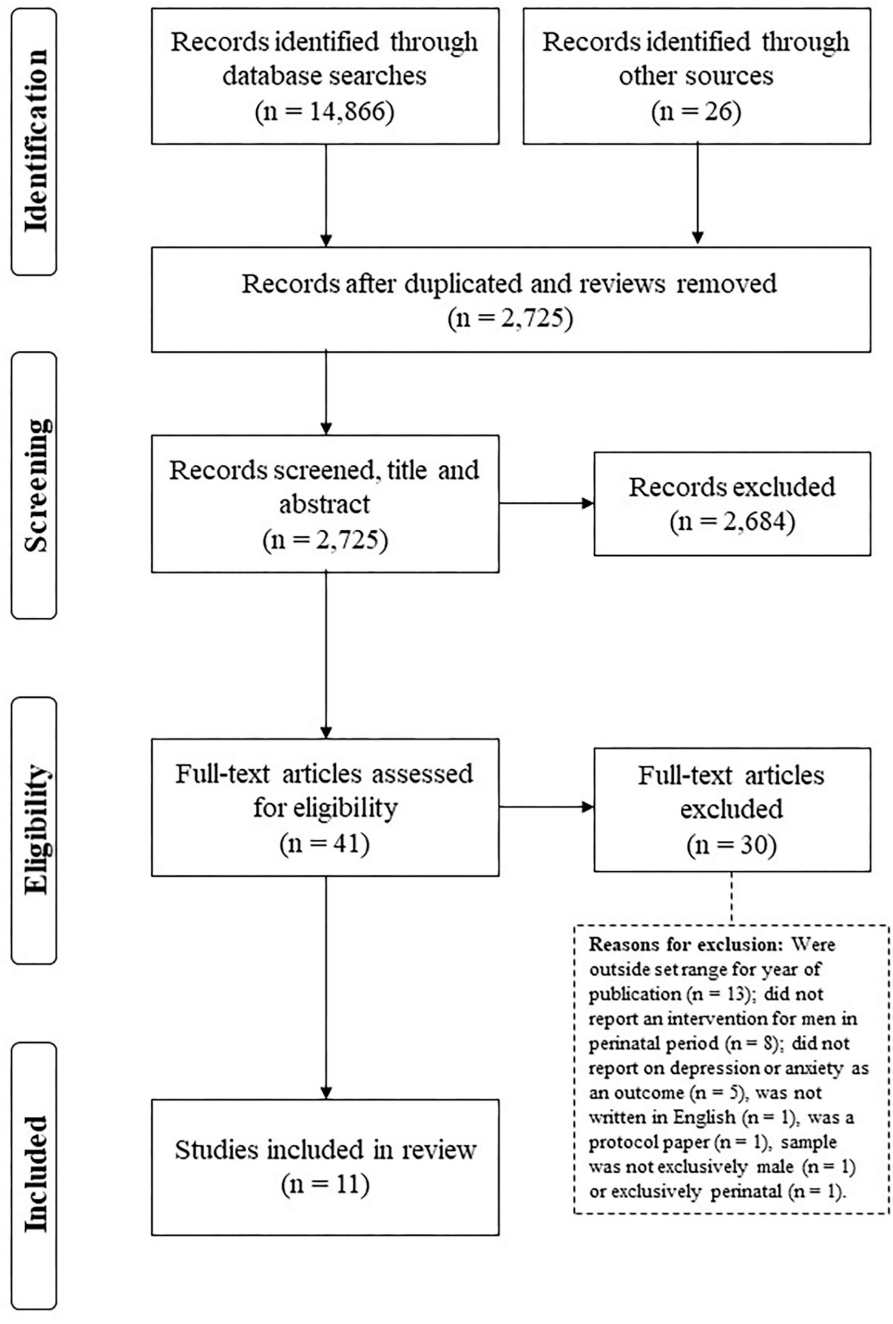
Study selection flow diagram.

The initial search identified 41 articles. After the removal of duplicates and inclusion of relevant studies identified via reference lists, 2,725 were screened by title and abstract. Following the removal of 2,684 ineligible articles, 41 were read in full before 30 studies were removed due to being considered ineligible with reasons outlined in Figure 1. Eleven studies were considered to meet the inclusion criteria and formed the final sample of studies for the mini-review. See Figure 1 for the flowchart of study selection.

Of these from the existing reviews, six were identified from the existing reviews (Table 1) and these plus five additional studies (Table 2) were located through the new literature search of electronic databases. Tables 1, 2 describe these 11 studies in detail.

**Table 1:** Published interventions reporting on men's perinatal depression or anxiety, 2015–2020.

**Identified in previous reviews**									
**References**	**Study design**	**Intervention program name; main elements and targets**	**Intervention timing and length**	**Intervention delivery format**	**Sample**	**Sample recruitment, engagement and retention**	**Mental health outcome measures**	**Timing of outcome measures**	**Intervention effects**
Castel et al. (2016)	Randomised Controlled Trial	Father-infant interactions, understanding of infant development to reduce parental stress, promotion of parents-infant triadic relationship to foster infant cognitive, motor, socio-emotional and behavioural development.	**Postnatal**: 22 sessions, home visits twice per month during first 4 months, followed by monthly consultations in neonatology ward up to 18 months	Face-to-Face. Triadic parent-infant Relationship Therapy (TRT), (based on attachment theory).	65 families with pre-term babies. **Intervention** ***N*** **=** **23** **Control** ***N*** **=** **19**	***N*** **=** **42 expecting fathers** included in final analysis. Recruited via partner couple. First 4 months were home-based.	Parenting Stress Index Short Form (**PSI-SF**) **EPDS** (>9 for father depression). **PTSD**. **DSM-IV** (for screening.)	**Baseline** (DSM-IV, PSI-SF, PTSD, EPDS) **3-Months** (PSI-SF, EPDS) **9-Months** (PSI-SF, EPDS) **(End-Point) 18-Months** (PSI-SF, PTSD, EPDS)	**Depression:** Significant 2.6-point difference observed in EPDS between intervention and control group at 18 months (*p* < 0.05). Control: At baseline 14.3% of fathers scored >9 in EPDS vs. 10.5% at 18 months. Intervention: At baseline 10.0% of fathers scored >9 in EPDS vs. 0% at 18 months (differences but not significant). **PTSD:** A significant−1.1.-point difference was observed for fathers of intervention group compared to control at 18 months (*p* < 0.001)
Daley-McCoy et al. (2015)	Cluster Randomised Controlled Trial	Content involved normalising adverse changes in relationship functioning reported by many couples on becoming parents and sharing potentially useful ways of managing these.	**Antenatal:** program consisted of 5 weekly, 2-h evening sessions.	Face-to-face. Low-intensity, psycho-educational program as adjunct to existing antenatal classes led by midwife.	Expectant fathers as part of couples expecting their first child. **Intervention** ***N*** **=** **39** **Control** ***N*** **=** **26**	***N*** **=** **37 expecting fathers** included in final analysis Recruited as a couple, via child services. Offered financial incentive. Extended intervention session by an additional 2-hr session to optimised attendance.	**EPDS**	**Baseline**, unknown gestation: (EPDS) **End-Point, 6-weeks Postpartum**: (EPDS)	**Depression:** Significant reduction in intervention group compared to control, (*p* = 0.023)
Shorey et al. (2017)	Randomised Controlled Trial	Home-but not Alone. Psychoeducation support to parents, featuring a database on role-specific educational contents, periodic push notifications to give timely information and asynchronous communication with healthcare professionals	**Postnatal**: Early stages	Digital deliver. Psychoeducational program via mobile-health application.	125 couples recruited, including 125 fathers. **Intervention** ***N*** **=** **63** **Control** ***N*** **=** **62**	***N*** **=** **125 fathers** included in final analysis. Delivered mobile phone app. Recruited via tertiary hospital, via couple. Phone app made information easy to access.	**EPDS**	**Baseline**: (EPDS) **End-Point**, 6 months postpartum: (EPDS)	**Depression**: No significant effect (*p* > 0.05).
Charandabi et al. (2017)	Single-Blind Randomised Controlled Clinical Trial	Prenatal lifestyle-based training included sleep health, nutrition, physical and sports activity, self-image and sexual problems. The training materials were presented by a male psychologist at the health centre office. 5–15 participants in each group session.	**Antenatal and Postnatal:** 24–28 weeks gestation and following 6 weeks postpartum. Weekly sessions were 60–90 min in length. Counselling calls were once/week, 10 min in duration (occurring between group sessions).	Face-to-face and Telephone. Two weekly group lifestyle-based training sessions, and telephone counselling sessions provided between sessions. All fathers of the intervention group were provided training booklet.	Spouses of pregnant women with gestational ages of 24–28 weeks. **Intervention** ***N*** **=** **62** **Control** ***N*** **=** **63**	***N*** **=** **125 fathers** included in final analysis Recruited via partners. Male psychologist was used to deliver intervention. Only 2 sessions due to fathers being busy.	**EPDS** **STAI**	**Baseline:** (EPDS, STAI) **+8 Weeks:** (EPDS, STAI) **End-Point, 6 Weeks Postpartum:** (EPDS, STAI)	**Depression:** At +8 Weeks, significant difference between intervention and control in reduction (*p* = 0.004). At 6 Weeks postpartum, significant difference between intervention and control in reduction (*p* = 0.001) **Anxiety:** At +8 Weeks, significant difference between intervention and control in reduction (*p* < 0.001). At 6 Weeks postpartum, significant difference between intervention and control in reduction (*p* < 0.001)
Huang et al. (2019)	Randomised Controlled Trial	Treatment group newborns placed in prone position onto bare chest of fathers, covered with clothes/blanket. Temperature set between 24 and 26 degrees Celsius.	**Postnatal:** Involved 30 min of skin-to-skin contact with father soon after caesarean delivery. Routine care taken to crib accompanied by father for 30 min.	Skin-to-Skin Contact.	108 fathers recruited. **Intervention** ***N*** **=** **54** **Control** ***N*** **=** **54**	***N*** **=** **100 fathers** included in final analysis Recruited from tertiary hospital via mothers. One session.	**SAS** (Self-Rating Anxiety Scale) **SDS** (Self-Rating Depression Scale)	**Baseline** (SAS, SDS) **End-poin**t, 30 min after treatment (SAS, SDS)	**Anxiety:** Lower in intervention group compared usual care (*p* < 0.05) **Depression:** Lower in intervention group compared usual care (*p* < 0.05)
Mihelic et al. (2018)	Randomised Controlled Trial	Baby Triple P, parenting intervention. Targets the key risk factors for poor child developmental outcomes identified in early infancy (i.e., parental mental health, couple adjustment, and parenting confidence and skill.)	**Antenatal and postnatal**: parenting program. 4 × 2 h face-to-face sessions, and 4 × individual postnatal telephone consultations.	Face-to-face and telephone.	112 couples recruited, resulting in 112 fathers recruited. **Intervention** ***N*** **=** **57 Control** ***N*** **=** **55**	***N*** **=** **107 fathers** included in final analysis Recruited via mother, high attrition rate. Mainly targeted mothers.	**EPDS** **DASS-21**	**Baseline**, pregnancy: (EPDS) **10 weeks' postpartum**: (EPDS) **End-Point**, 6 months' postpartum: (EPDS)	**Depression:** No significant effect (*P* > 0.05)

**Table 2:** Published interventions reporting on men's perinatal depression or anxiety, 2015–2020.

**Identified by this Mini-Review**									
**References**	**Study design**	**Intervention program name; main elements and targets**	**Intervention timing and length**	**Intervention delivery format**	**Sample**	**Sample recruitment, engagement and retention**	**Mental health outcome measures**	**Timing of outcome measures**	**Intervention effects**
Cano Giménez and Sánchez-Luna (2015)	Prospective Study	Parent/mother–infant interaction Parent/mother–healthcare staff interaction Coping with the new unexpected situation	**Postnatal**: 15 days	Face-to-face by Psychologist Intervention adapted by Lester et al. Interdisciplinary characteristics with participation and collaboration of all staff working in the NICU including physicians, nurses & nurse assistants.	Mothers and Fathers with new baby admitted to NICU with congenital heart defect or perinatal hypoxic-ischemic encephalopathy (at least 4-week NICU admission). **Intervention** ***N*** **=** **25** **Control** ***N*** **=** **29**	***N*** **=** **54 fathers** included in final analysis. Recruited via partner, no engagement strategies.	**Parental Stressor Scale-NICU**, **ISRA** **BDI** **EPDS**	**Baseline** 3 days after NICU admission (PSS-NICU, ISRA, BDI, EPDS) **End-point**: 15 days after NICU admission (PSS-NICU, ISRA, BDI, EPDS)	**Anxiety**: 0% in intervention compared to 89.6% in control (*p* = 0.001) at end-point. **Depression** (BDI): 20% in intervention with mild depression compared to 100% in control group with mild-moderate depression (*p* < 0.001) at end-point. **Depression** (EPDS): 24% in intervention group probable depression, compared to 89.7% in control group (*p* < 0.001) at end-point. 44% of intervention group without risk of depression, 0% in control (*p* < 0.001), at end-point.
Edward et al. (2019)	Single blinded Randomised Controlled Trial	General PND information, statistics of paternal PND, the EPDS and instructions on how to complete and score it, and advice regarding referral to their General Practitioner (GP) should the participant be distressed or concerned about their EPDS score.	**Antenatal:** One-time self-screening tool for mother and father and information pamphlet.	Delivered by pamphlet Self-screening and referral pathway that was a one- page (A4 size).	70 Expectant Fathers recruited from 140 couples **Intervention** ***N*** **=** **35** **Control** ***N*** **=** **35**	***N*** **=** **31** expecting fathers included in final analysis Recruited via couple. High attrition. Phone call follow-up occurred.	**EPDS** **Kessler-10 (K-10)**	**Baseline** (EPDS, K-10) **End-Point**, 12 months (EPDS, K-10)	**Depression:** No significant effects (*p* < 0.05)
Herman (2020)	Pre-Post Quasi-Experimental Pilot Study	“PREParing for Parenthood (PREP)” antenatal class. Psychoeducational, partner inclusive focusing on depression reduction, stress management and enhancing co-parent relationship.	**Antenatal**: classes between 10 and 20 weeks gestation. Six-week intervention.	Face-to-face. Six sessions taught by paraprofessionals in a community setting (optional home visits for usual care).	46 couples recruited. **Intervention** ***N*** **=** **24** **Control** ***N*** **=** **22**	***N*** **=** **37** expecting fathers included in final analysis. Recruited via couple, not only male partners. Honorarium provided. Used male and female instructors.	**CES-D** (Centre for Epidemiologic Studies Depression Scale) **PSS** (Perceived Stress Scale).	**Baseline**, **beginning of 2nd trimester:** (CES-D, PSS) **End-Point**, **+8–10 weeks after Baseline** (CES-D, PSS)	**Stress:** Significant reduction in intervention compared to control (*p* = 0.031). **Depression:** No significant effects (*p* > 0.05).
Missler et al. (2020)	Randomised Controlled Trial	Targeting sensitive responsiveness, adapting to the parental role, attending to own needs. Crying patterns, feeding and sleeping.	**Antenatal**: Program occurred, between 36- and 34-weeks gestation.	A booklet, a video, a home visit and a telephone call.	From 138 pregnant women, 96 partners were recruited. **Intervention** ***N*** **=** **31 Control** ***N*** **=** **38**	***N*** **=** **69 fathers** included in final analysis. Recruited via couple, fathers recruited incidentally.	**PSI** (Parenting Stress Index), **EPDS** **HADS**	**Baseline**, 26–34 weeks: (PSI, EPDS, HADS) **34–36 weeks**: (PSI, EPDS, HADS) **6 weeks** postpartum: (PSI, EPDS, HADS) **End-Point**: 10 weeks postpartum: (PSI, EPDS, HADS)	**Depression:** No significant effect (*p* > 0.05). **Anxiety:** No significant effect (*p* > 0.05).
Setodeh et al. (2017)	Pre-Post Experimental Study	Face-to-face training sessions.	Antenatal sessions between 28 and 34 weeks. 4 × 90-min sessions once a week on maternal-foetal attachment.	Fathers were trained regarding attachment skills.	150 pregnant women's spouses recruited. **Intervention** ***N*** **=** **75** **Control** ***N*** **=** **75**	*N* = 150 expecting fathers included in final analysis. Recruited through pregnant women.	**SBS** (Spiel Berger Scale) (Anxiety).	**Baseline**, 28–34 weeks: (SBS) **End-Point**, 1 month after intervention: (SBS).	**Anxiety:** Reduced in intervention group (*p* = 0.008) compared to control.

### Study Characteristics

Although this mini-review did not apply a formal framework of quality assessment, it is of note that eight out of 11 studies were Randomised-Controlled Trials (RCTs), the gold-standard in the hierarchy of evidence for the evaluation of health outcomes. Of the three remaining studies, one was a pre-post experimental study (Setodeh et al., 2017), one was a prospective study (Cano Giménez and Sánchez-Luna, 2015) and one was a pilot study (Herman, 2020). Sample sizes ranged from *n* = 42 to *n*= 146. Three studies sampled specific populations, including: one study focused on mothers and fathers with infants who were admitted to the Neonatal Intensive Care Unit (Cano Giménez and Sánchez-Luna, 2015), one study included mothers and fathers with preterm babies (Castel et al., 2016), and one study sampled fathers with newborns following caesarean section (Huang et al., 2019). Of the remaining studies, one study sampled primiparous men (Daley-McCoy et al., 2015), four studies sampled expectant couples with no limits on parity (Setodeh et al., 2017; Edward et al., 2019; Herman, 2020; Missler et al., 2020), one study sampled couples in the postpartum with no limits on parity (Shorey et al., 2017), and two studies sampled expectant couples with intervention implementation throughout pregnancy and the postpartum (with no limits on parity) (Charandabi et al., 2017; Mihelic et al., 2018). Timing of delivery varied among studies, with most occurring either exclusively during the antenatal (Daley-McCoy et al., 2015; Setodeh et al., 2017; Edward et al., 2019; Herman, 2020; Missler et al., 2020) or postnatal period (Cano Giménez and Sánchez-Luna, 2015; Castel et al., 2016; Shorey et al., 2017; Huang et al., 2019). Two interventions were delivered across both time periods (Charandabi et al., 2017; Mihelic et al., 2018).

### Summary of New Individual Studies

We identified five new studies (Cano Giménez and Sánchez-Luna, 2015; Setodeh et al., 2017; Edward et al., 2019; Herman, 2020; Missler et al., 2020) in addition to those included in previous reviews. Cano and Giménez recruited fathers as part of couples with infants admitted to the neonatal intensive care unit (NICU). Parents underwent a tailored five-step intervention delivered by a psychologist. Fathers in the intervention group had significantly lower levels of anxiety and depression after 15 days, compared to the control group. Setodeh et al. investigated the effect of four 90-min fathers' attachment training sessions on anxiety and observed lower anxiety scores at follow-up.

Edward et al. delivered a self-screening tool and referral pathway pamphlet as part of an RCT. They did not observe any statistically significant differences between groups for depression at the end of the study. Herman et al. reported on a quasi-experimental study of a 6-week psycho-educational intervention aiming to reduce depression in expectant parents in early pregnancy. No significant effects were observed for depression in fathers. Missler et al.s' intervention was also delivered antenatally, during weeks 26 and 34, as part of an RCT. Their intervention consisted of a booklet, video, home visit and phone call and again, there were no interventional effects on fathers' depression at follow-up.

### Types of Interventions and Focus

A large proportion of interventions incorporated counselling or therapy (Cano Giménez and Sánchez-Luna, 2015; Castel et al., 2016; Charandabi et al., 2017; Mihelic et al., 2018) or psychoeducation as part of an antenatal or postnatal education program (Cano Giménez and Sánchez-Luna, 2015; Daley-McCoy et al., 2015; Castel et al., 2016; Charandabi et al., 2017; Shorey et al., 2017; Mihelic et al., 2018; Edward et al., 2019; Herman, 2020; Missler et al., 2020), while massage was the focus of Huang and colleagues (Huang et al., 2019). Of the five studies offering therapy or counselling, the focus was varied and included parent-infant interaction/attachment (Cano Giménez and Sánchez-Luna, 2015; Castel et al., 2016; Setodeh et al., 2017), men's self care/self image (Shorey et al., 2017), and parenting (Mihelic et al., 2018).

Most of the interventions were couple-focused, with only one (Huang et al., 2019) focusing exclusively on fathers. Couple-focussed programs targeted couple- and father-baby relationships and interactions, coping strategies, physical contact (partner and/or baby), problem solving and emotion self-management. The father-focussed program provided information/education, and strategies on how to better support mothers.

### Assessment and Outcome Measures of Men's Depression and Anxiety

No studies intentionally sought to recruit men with perinatal depression or anxiety.

Across studies, a variety of different depression and anxiety measures were reported. The Edinburgh Postnatal Depression Scale (EPDS) was the predominant psychometric outcome measure reported (Cano Giménez and Sánchez-Luna, 2015; Daley-McCoy et al., 2015; Castel et al., 2016; Charandabi et al., 2017; Shorey et al., 2017; Mihelic et al., 2018; Edward et al., 2019; Missler et al., 2020). One study reported on anxiety using the State-Trait Anxiety Inventory (STAI) (Charandabi et al., 2017) and another using the Spiel Berger Scale (SBS) (Setodeh et al., 2017) while another study reported on depression using the Centre for Epidemiological Studies Depression Scale (CES-D) (Herman, 2020). No studies reported a presence of diagnosed depression at baseline. When reported, baseline levels were well-below the threshold for probable depression. Two studies did not report on depression or anxiety at baseline (Cano Giménez and Sánchez-Luna, 2015; Shorey et al., 2017). Despite this, five studies reported a positive effect on depression (Daley-McCoy et al., 2015; Castel et al., 2016; Charandabi et al., 2017) and/or anxiety (Cano Giménez and Sánchez-Luna, 2015; Charandabi et al., 2017; Huang et al., 2019).

### Engagement Strategies

Few studies evaluated the effectiveness of engagement or retention strategies.

Initial engagement of men spanned various settings, often through secondary contact via pregnant partners, ranging from prenatal programs and hospital settings (including one neonatal intensive care unit) to local advertising and health service referrals (such as general practitioner or ultrasound clinic).

Despite the range of engagement strategies used, no studies provided a concrete evaluation of their effectiveness. Whilst just over half of the studies reported no attrition at follow-up (Cano Giménez and Sánchez-Luna, 2015; Castel et al., 2016; Charandabi et al., 2017; Setodeh et al., 2017; Shorey et al., 2017; Missler et al., 2020), there were no discernible differences in design or program type between these and studies with higher attrition rates. The remaining studies had attrition rates ranging from 4 to 55%. Whilst no clear patterns across studies were observed, home-based intervention was suggested by authors as a method to improve adherence (Castel et al., 2016). Financial incentives may also play a role (Daley-McCoy et al., 2015). Incentives including free dinners, raffles for attendance, honorariums, and money for transport were reported by authors as likely to contribute to strong attendance rates (Herman, 2020).

Across the different study designs and methodologies, there were some commonalities in recruitment procedures. Recruitment of fathers indirectly through their female partners was very common. Whether this improved engagement is unclear. For example, one study (Mihelic et al., 2018) argued this method in their study may have been responsible for low father participation rate in a group triple P program and advocated for separate father sessions.

Other incentives to combat sample attrition included utilising supportive technology to circumvent potential barriers, such as telephone calls, text messaging, the internet and a mobile phone app. Male psychologists were also utilised, perhaps proving beneficial for participant retention (Seidler et al., 2018). Some studies attempted to increase retention by keeping the intervention time-commitment as low as possible. Other studies reported offering more of the popular aspects of their intervention such as psychoeducation about newborns or birth, subsequently minimising the focus on other topics, such as targeting the co-parenting relationship.

## Discussion

Due to the prevalence of mental health problems in expectant and new fathers, there is a need for interventions targeting mental health difficulties in fathers, primarily depressive or anxious symptoms. Yet the three most recent systematic reviews found that few such treatment studies exist.

We aimed to conduct an update of the literature and provide a narrative synthesis of peer reviewed articles published between 2015 and 2020 that report trials, feasibility studies or pilot studies of interventions that reported on fathers' perinatal depression or anxiety. A secondary purpose of the mini- review was to describe any promising evidence of key implementation, engagement and retention strategies.

Only eleven publications were found to satisfy search criteria, six of which were included in previous reviews. Rominov et al. found that psychosocial interventions and massage-technique interventions, but not couple-based interventions, showed some significant effects on depression and anxiety symptoms benefitting men. It was concluded that there was a need for improved methodological quality in this field and that none of the interventions included active treatment targeting clinical levels of psychological distress in perinatal fathers. Suto et al. concluded that there was insufficient evidence to suggest prenatal childbirth education for partners of pregnant women protects against paternal postnatal depression and anxiety. Goldstein et al. found none of the included interventions exclusively focussed on paternal perinatal depression but targeted the couple or parent-infant relationship. The lack of active treatments for diagnosed cases of paternal perinatal depression was also highlighted. Although we identified 5 new studies, our updated findings confirm a continuing lack of interventions targeting perinatal depression or anxiety in fathers, in particular when compared to the large body of literature on maternal perinatal depression and anxiety interventions. Congruent with articles identified by previous reviews, the majority of programs took a universal approach, not explicitly addressing paternal perinatal anxiety and/or depression. Compared to all of the six studies included in previous reviews, only two of the newly identified articles in this mini-review were RCTs. No studies required participants to screen positive for anxiety or depression and none of them targeted men who were depressed or anxious. Only two were able to show an improvement in depressive (Cano Giménez and Sánchez-Luna, 2015) and/or anxiety (Setodeh et al., 2017) symptoms, compared to four of the articles included in previous reviews (Daley-McCoy et al., 2015; Castel et al., 2016; Charandabi et al., 2017; Huang et al., 2019).

The ability to draw conclusions on the effectiveness of the interventions identified is challenged due to a number of factors. First, the studies reviewed here pertained largely to universal programs assisting fathers and mothers in the transition to parenthood, with no studies designed explicitly to treat existing depressive or anxiety disorders in men. Further, no studies required participants to undertake a clinical interview for diagnosis or screen positive for anxiety or depressive symptoms on entry. In fact, in some studies, depression formed part of the exclusion criteria (Castel et al., 2016; Charandabi et al., 2017; Shorey et al., 2017). Second, paternal depression or anxiety outcomes usually formed part of secondary outcome analyses or at times were reported incidentally as a part of an intervention targeting new or expectant mothers.

The comparative lack of intervention for men compared to women in the perinatal period is reflected in national practise guidelines in Australia and the UK. While these clinical guidelines are extensive there are no recommendations for paternal mental health during the perinatal period, beyond recommendation for further research. By contrast, clinical guidelines provide recommendations for structured individual psychological therapy (e.g., CBT or IPT) for treatment of depression for women during the perinatal period (Howard et al., 2014; Austin et al., 2017).

Our results therefore provide an updated confirmation of the broad conclusions reached in the previous foundational reviews by Rominov et al. (2016), Suto et al. (2017), and Goldstein et al. (2020). Namely, that despite the increased interest and accumulating research evidence in paternal mental health in recent years, this has not translated into a substantial research literature aimed at evaluating interventions addressing paternal perinatal depression and anxiety. Specifically, there is an almost total absence of treatment programs evaluated in RCTs designed to treat fathers with a perinatal depressive or anxiety disorder.

## Implications for Implementation

Despite the heterogeneity of study methodologies, some tentative descriptive analysis of findings that may be relevant to implementation and development of future programs is possible.

First, it is worth noting that most reviewed studies used the EPDS as a depression measure, indicating this has become a commonly used research measure of not only maternal, but also paternal, depressive symptoms.

Secondly, whilst eight out of 11 studies were randomised controlled trials (RCTs), none of the RCTs were large studies, and future replication in adequately powered trials would be needed before any definitive conclusions on efficacy could be drawn. The study populations in the studies identified by this mini- review did not, on average, have elevated levels of depressive or anxiety symptoms that would be of clinical concern. Therefore, even where statistically significant improvements in depression and anxiety scores were reported, the clinical meaningfulness of these findings and their generalisability for depressed and anxious populations of perinatal men is doubtful.

Nevertheless, it may be useful to make the following observations about studies which reported improvements in either depression scores (five studies) or anxiety scores (four studies). In terms of positive paternal mental health outcomes, the largest gains were seen in interventions which incorporated therapy/counselling components, antenatal psycho-education or baby massage. At the very least, taken together, the results of these studies suggest that symptoms of anxiety as well as depression could potentially be amenable to positive change through interventions designed for perinatal men.

### The Possible Value of Indirect Methods to Engage Men

None of the reviewed studies aimed to directly identify and treat clinical levels of perinatal depression or anxiety in men. However, this might not be the only viable model for addressing paternal depression.

Using a range of more indirect approaches could, potentially, help reach a depressed/anxious population by dealing first with other salient issues of concern for new fathers. Such an approach could serve to deliver a pre-emptive or preventive support for paternal mental health. For example, involving partners in family and couple focussed programs aimed at enhancing those relationships may be a viable way to engage men initially and set a supportive context for recognising paternal mental health issues as and when they emerge. This could provide a more acceptable foundation for offering targeted treatment programs for those men who need them, should clinical levels of depression or anxiety arise.

Similarly, several interventions adopted strategies to promote fathers' attachment with their infants and support the partner relationship and these appeared to have some positive effects for fathers' wellbeing. However, as well as enhancing well-being, it may also be useful to explore whether such a focus assists in engaging men with perinatal depression or anxiety in accessing and adhering to support programs or to treatment programs.

In general, such indirect approaches based on framing content around transition to fatherhood and the new relationships between father and baby may be more acceptable to men than content that is directly and explicitly about identifying and treating mental disorders. For example, in a recent DELPHI study (Domoney et al., 2020) about developing interventions for paternal perinatal depression, the consensus was that better recruitment, retention and engagement may be attained by using a “strengths-based” approach in presenting treatment content.

Additional findings, outside of the parameters of the current mini-review, may also inform potential approaches to increasing engagement with paternal mental health supports. For example, fathers recount the benefits of programs that encourage social contact with other fathers (Herman, 2020), helping them feel less isolated and share in discussions about fatherhood (Seymour et al., 2020).

There is some evidence (Giallo et al., 2017) that facilitators to male engagement include accessibility of programs; and barriers may include time commitments and the need to travel. Internet delivery could potentially fit with these preferences and allow men to feel empowered to drive their own treatment and recovery. Indeed, in a survey of 154 Australian fathers of young children (Parry et al., 2019), common attitudinal barriers to engaging with help-seeking included beliefs about self-reliance in managing one's own problems and suggested that men would prefer internet-based supports.

## Limitations

As previously described, there was a significant level of heterogeneity across the methodologies in the studies captured by this mini-review. It is therefore difficult to proceed to a formal analysis of key parameters such as efficacy. In addition to insufficient reporting of study designs and differences in timing of program delivery, the greatest limitation to this and preceding reviews is the general absence of studies evaluating programs designed specifically to treat paternal perinatal clinical disorders (such as depression and anxiety). The available evidence allows only a speculative consideration of what might potentially be the most effective elements and ways to configure such a treatment program in the real world.

## Future Directions and Conclusions

This mini-review highlights a number of significant gaps and potentially productive avenues for future research.

Of greatest note was the absence of evidence-based psychological treatments for diagnosed depression and anxiety in men. Such programs have proven to be effective for perinatal depression in women (Dennis and Hodnett, 2007; Milgrom et al., 2011), with CBT and interpersonal therapy (IPT) extensively investigated. Given the relative absence of psychological interventions (*n* = 2) amongst the work reviewed here, further research will be needed to ascertain whether such interventions can be effective in a depressed population of perinatal men.

While reviewed studies offered some evidence that a range of universal programs have some potential to improve symptoms of anxiety and depression, whether such interventions can be effective in a real-world population of depressed and anxious perinatal men has not yet been demonstrated.

Nevertheless, such programs may provide useful indirect entry points for engaging and subsequently recognising men who may be experiencing clinical levels of depression and anxiety warranting direct treatment. Engagement strategies to engage men in seeking support for their mental health in the perinatal period therefore requires further research, including the possible benefits of online delivery. Whilst face-to-face programs offer a supportive environment, it is possible this mode of delivery adds others barriers to engagement as perinatal fathers are typically time-poor, often with increased work commitments (Bayley et al., 2009).

To ensure that appropriate interventions are developed, further research is needed to understand how men experience distress in the perinatal period, including both anxiety and depression. This should include the overlap of symptomatology and co-morbidity of depressive and anxiety disorders and further research on how their manifestations, such as anger, are associated with difficulties in the couple relationship, the co-parenting relationship, and the father-infant relationship (Macdonald et al., 2020, 2021).

## Author Contributions

AR, JE, AG, and JM were responsible for the design of the work and agreed upon the final search terms. AR and JE reviewed titles and abstracts for inclusion, with all authors AR, JE, BW, AG, and JM involved in screening manuscripts for eligibility, interpretation of findings, drafting the article, critically reviewing the article, and have approved of the version that has been submitted. All authors contributed to the article and approved the submitted version.

## Funding

This review was supported and made possible by the Ian Potter Foundation, Perpetual IMPACT Philanthropy and Men of Malvern. We gratefully acknowledge their contributions.

## Conflict of Interest

The authors declare that the research was conducted in the absence of any commercial or financial relationships that could be construed as a potential conflict of interest.

## Publisher's Note

All claims expressed in this article are solely those of the authors and do not necessarily represent those of their affiliated organizations, or those of the publisher, the editors and the reviewers. Any product that may be evaluated in this article, or claim that may be made by its manufacturer, is not guaranteed or endorsed by the publisher.
